# Comparative optimization of overcurrent relay coordination in DG-integrated distribution networks: water cycle algorithm versus genetic algorithm and big bang–big crunch

**DOI:** 10.1038/s41598-026-43242-z

**Published:** 2026-03-27

**Authors:** Reda E. Mohamed, Saber M. Saleh, Ahmad G. Ahmad

**Affiliations:** Electrical Engineering Department, Faculty of Engineering, Fayoum University, Fayoum, 63514 Egypt

**Keywords:** Distributed generation, Overcurrent relay coordination, Dual-setting overcurrent relays, Metaheuristic optimization, Water cycle algorithm, Genetic algorithm, Big bang–big crunch, Energy science and technology, Engineering, Mathematics and computing

## Abstract

The increasing penetration of distributed generation (DG) has significantly complicated protection coordination in modern distribution networks by introducing bidirectional power flows and variable fault current levels. These challenges become more pronounced under different operating modes, particularly in islanded operation, where reduced fault current levels place severe constraints on overcurrent relay (OCR) coordination. This paper presents a comparative assessment of metaheuristic optimization techniques for coordinating overcurrent protection in DG-integrated distribution networks. Two distribution systems with different topological characteristics are examined. A radial 9-bus system is analyzed under three operating modes: conventional grid-connected operation without DG, DG-integrated grid-connected operation, and islanded operation. In contrast, a meshed 30-bus distribution system is evaluated exclusively under islanded operation to represent the most demanding coordination conditions, where fault current support is limited to inverter-based DG sources. Relay coordination is formulated as a constrained optimization problem aimed at minimizing total relay operating time while satisfying coordination time interval (CTI) requirements. Three metaheuristic optimization algorithms are applied under identical protection models and fault conditions: the Genetic Algorithm (GA) as a conventional reference approach, and the Water Cycle Algorithm (WCA) and Big Bang–Big Crunch (BB-BC) algorithm as more recent techniques. Short-circuit calculations are performed for three-phase faults in accordance with IEC 60909 standards. The results indicate that all investigated algorithms successfully achieve coordinated relay settings under the examined operating modes.

## Introduction

Modern distribution networks are undergoing a fundamental transformation driven by the increasing penetration of DG, particularly inverter-based renewable energy resources. While this transition offers well-recognized environmental and economic benefits, it has introduced substantial challenges to conventional protection schemes that were originally designed for passive, radially operated systems^1,2^.

Conventional OCR coordination in distribution networks relies on the assumption of unidirectional power flow, where fault currents originate from the main grid and propagate downstream toward load buses. Under this assumption, selective coordination between primary and backup relays can be achieved by appropriately grading relay operating times based on fault current magnitudes and predefined CTIs^3,4^. However, the integration of DG invalidates this assumption by introducing additional fault current sources within the network, leading to bidirectional fault current flows, altered short-circuit levels, and increased sensitivity of protection settings to network operating modes^5,6^.

In DG-integrated distribution systems, both the magnitude and direction of fault currents depend strongly on the number, type, and location of active generators, as well as on the operating mode of the network. These variations can result in several well-documented protection issues, including protection blinding, false tripping, sympathetic tripping, and loss of selectivity, which collectively degrade system reliability and may lead to unnecessary service interruptions. The problem becomes more pronounced in networks with high DG penetration and in configurations where meshed or looped structures are employed^7–10^.

Directional and Dual Setting Over Current Relays have been widely adopted to mitigate some of these challenges. By allowing separate settings for forward and reverse fault directions, such relays enhance selectivity in networks experiencing bidirectional fault currents. Nevertheless, the use of directional and dual-setting relays significantly increases the complexity of the coordination problem. The number of coordination constraints grows substantially, and relay performance becomes highly sensitive to network topology, fault location, and operating mode. As a result, conventional trial-and-error coordination approaches become impractical, particularly in systems with high DG penetration and multiple operating modes^11,12^.

Early research addressing these challenges primarily focused on analyzing the impact of DG on fault current characteristics and documenting coordination failures under different operating modes^13,14^. While these studies provided valuable insight into the nature of the problem, they largely relied on offline analyses and manual relay setting adjustments, which are impractical in modern distribution networks where DG output and system topology may change frequently. To overcome these limitations, adaptive protection schemes based on communication-assisted relays were subsequently proposed, enabling relay settings to be updated in real time according to operating modes^15,16^. However, such schemes typically require extensive communication infrastructure and introduce additional concerns related to latency, reliability, and cybersecurity^17,18^.

As an alternative, optimization-based relay coordination approaches employing metaheuristic algorithms have gained considerable attention. These methods are well suited to relay coordination problems due to their ability to handle nonlinear objective functions and discrete relay settings without requiring gradient information^19–21^. GA were among the earliest metaheuristics applied in this context and demonstrated satisfactory performance in minimizing relay operating times in relatively simple radial networks^22,23^. Nevertheless, in systems with high DG penetration, where coordination constraints increase significantly, GA has been reported to suffer from premature convergence and difficulty in satisfying all coordination requirements^24,25^. This limitation motivated the development of hybrid techniques, such as GA–NLP and GA–IPM, which combine global search capabilities with deterministic refinement methods to improve solution quality, albeit at the expense of increased computational complexity^29,30^.

Particle Swarm Optimization (PSO) has also been explored as an alternative, offering faster convergence characteristics in some studies. Several PSO variants and hybrid PSO-based approaches have been evaluated on IEEE benchmark systems, often demonstrating improved computational efficiency compared to standard GA implementations^26,27^. However, PSO performance has been reported to be sensitive to parameter selection, with reduced robustness observed under islanded operating modes characterized by lower fault current levels.

More recent research has investigated newer nature-inspired algorithms for microgrid and DG-dominated protection applications. The WCA, originally introduced by Eskandar et al.^28^, has shown promising performance for relay coordination in microgrids experiencing frequent operating mode transitions^29,30^. Similarly, the BB-BC algorithm has been applied to relay coordination problems with encouraging results^31^. Nevertheless, comprehensive comparative evaluations between these recent algorithms under identical protection models and operating conditions remain limited.

In parallel with advances in optimization methods, developments in relay technology have contributed to enhanced protection flexibility. DOCRs allow independent forward and reverse settings, which is particularly advantageous in meshed DG-integrated networks where fault current direction depends on the operating status of DGs^32,33^. Enhanced relay characteristics, such as time–current–voltage (TCV) tripping schemes, have also been proposed to improve coordination performance under varying fault impedances and DG contributions^34^. Furthermore, the increasing penetration of inverter-based renewable energy sources introduces additional complexity due to their inherent current-limiting behavior during fault conditions, particularly under islanded operation^35–37^.

Among the various operating modes of DG-integrated distribution systems, islanded operation represents the most demanding mode from a protection coordination perspective. In this mode, the main grid is disconnected and fault current contributions are limited to inverter-based DG units, resulting in substantially reduced fault current levels and narrower coordination margins. Under such conditions, maintaining reliable primary–backup discrimination using conventional coordination practices becomes increasingly challenging^38–41^. Despite its practical importance, systematic comparisons of metaheuristic optimization algorithms under identical protection models and stressed islanded operating modes remain limited, particularly for systems employing directional and dual-setting relays.

Accordingly, this study focuses on the systematic investigation of OCR coordination in DG-integrated distribution networks under different operating modes, with particular emphasis on islanded operation. By considering both radial and meshed network topologies and applying identical protection models and fault scenarios, this work aims to provide a consistent comparative assessment of the performance and robustness of selected metaheuristic optimization techniques.

## System description and research methodology

This study adopts a systematic framework for OCR coordination in distribution networks with DG, combining detailed network modelling, short-circuit analysis, and optimization-based relay setting determination. The overall methodology is designed to ensure a fair and consistent comparison between the investigated optimization algorithms under identical protection models and operating modes.

### Overview of research framework

The coordination process is implemented through an integrated simulation environment in which network modelling and fault analysis are performed using ETAP software, while the optimization algorithms are executed in MATLAB. ETAP is employed to model the distribution systems, calculate load flow conditions, and perform short-circuit studies in accordance with IEC 60,909 standards. MATLAB is used to formulate and solve the relay coordination optimization problem using the selected metaheuristic algorithms. The primary objective of the adopted framework is to determine optimal relay settings that minimize the total operating time of primary relays while ensuring proper coordination with their associated backup relays. To achieve this objective, identical fault scenarios, relay characteristics, and coordination constraints are applied to all investigated algorithms, allowing their performance to be compared on a consistent basis.

### Test system configurations

Two distribution systems with different topological characteristics are considered in this study in order to evaluate the performance of the optimization algorithms under both relatively simple and highly constrained coordination environments.

#### 9-Bus radial system

**Fig. 1:**
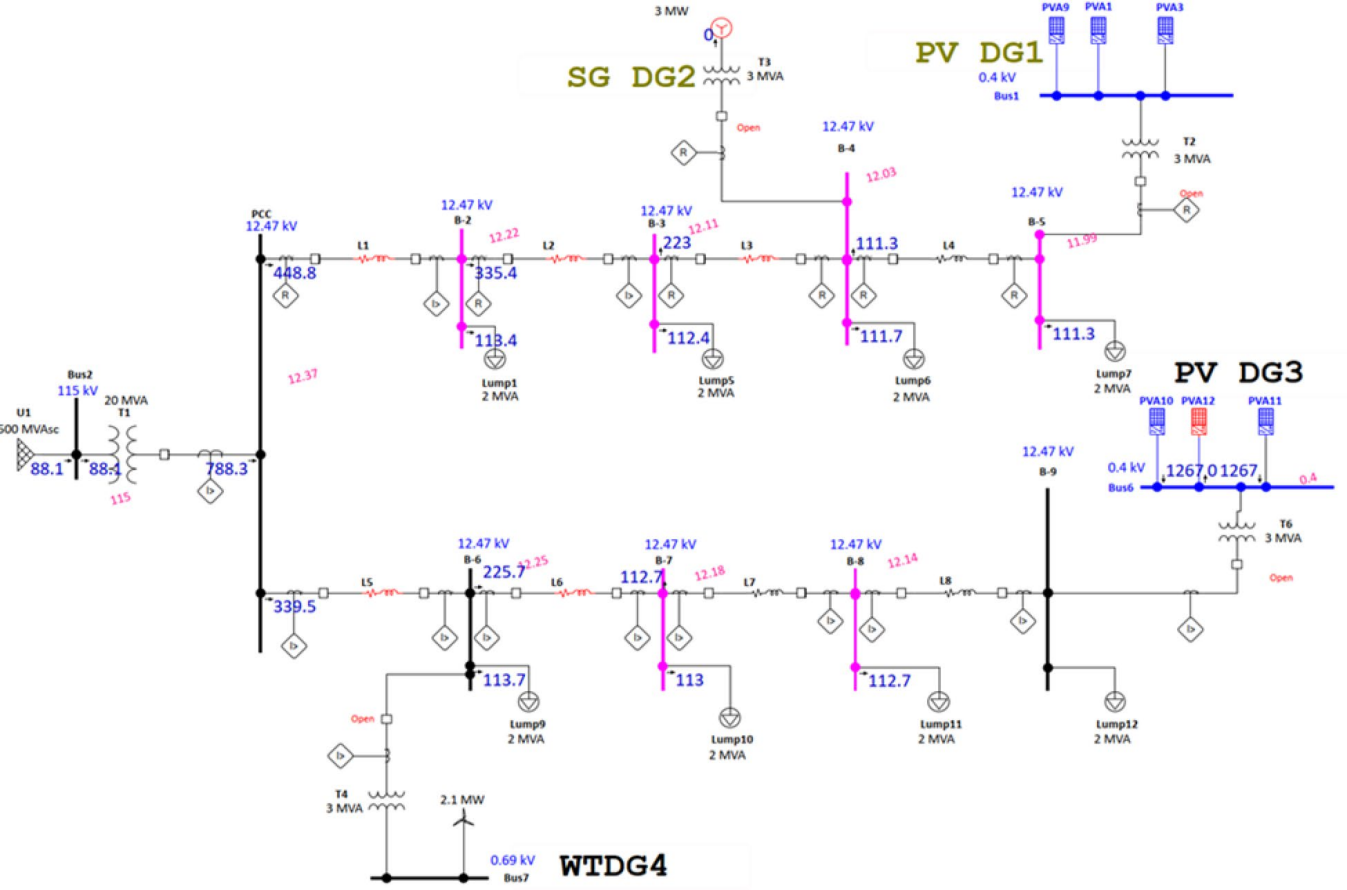
Single-line diagram of the 9-bus radial distribution system with DG.

Figure 1 illustrates a DG-connected Canadian urban radial 9-bus distribution network modelled in ETAP and operating at a nominal voltage of 12.49 kV^42,43^. The system includes four DG units of different technologies: a 3 MVA synchronous generator at bus 4, two photovoltaic units each rated at 3 MVA at buses 5 and 9, and a 2.471 MVA wind turbine generator at bus 6, resulting in varying fault current contributions under different operating modes. The protection scheme consists of 21 OCRs arranged to provide coordinated primary and backup protection throughout the network. Relay settings, including CTR, pickup currents *I*​p, and plug settings (PS), are determined based on load flow analysis and are summarized in Table 1.Coordination performance is evaluated under three operating modes: conventional grid-connected operation without DG, grid-connected operation with full DG integration, and islanded operation. Three-phase faults are applied at eight strategic locations (F1–F8) along the feeders to assess selectivity.

**Table 1:** Pickup current values of OCR in the 9-bus system.

Relay ID	CTR	PS	I_pickup (A)
OCR1	400	0.5	200
OCR2	400	0.625	250
OCR3	400	1.0	400
OCR4	400	1.25	500
OCR5	400	0.5	200
OCR6	400	0.625	250
OCR7	400	0.5	200
OCR8	400	1.25	500
OCR9	400	0.25	100
OCR10	400	0.25	100
OCR11	400	0.25	100
OCR12	400	0.25	100
OCR13	400	0.25	100
OCR14	400	0.25	100
OCR15	400	0.25	100
OCR16	400	0.25	100
OCR17	1000	1.0	1000
OCR18	400	0.5	200
OCR19	400	0.5	200
OCR20	400	0.5	200
OCR21	400	0.5	200

#### 30-Bus mesh system

**Fig. 2:**
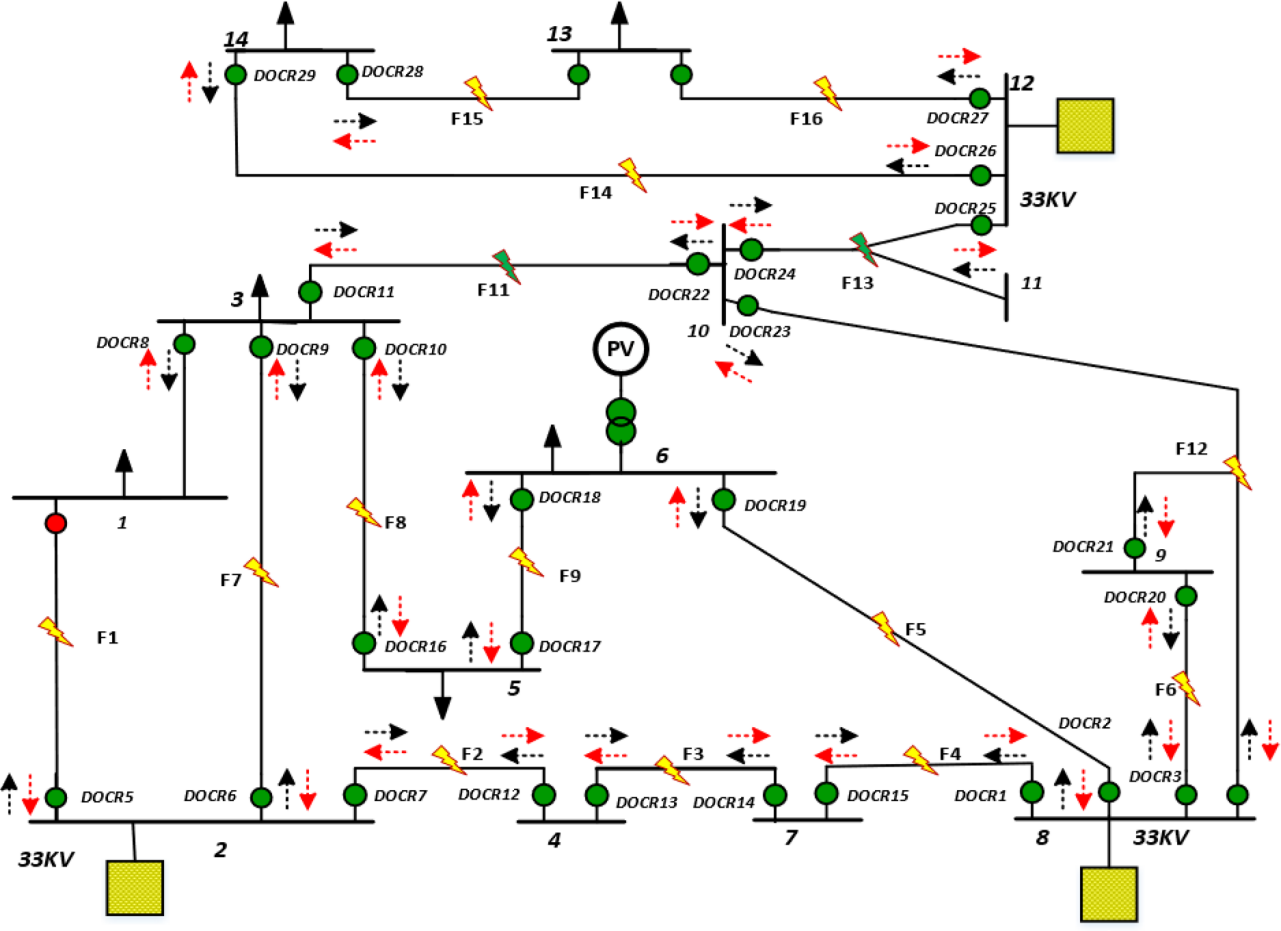
Single-line diagram of the 30-bus mesh distribution system with DOCRs.

Figure 2 illustrates the second test system, which is a meshed 30-bus distribution network modelled in ETAP and reported in^44,45^. The system is analyzed exclusively under islanded operation, where it is completely decoupled from the upstream utility grid, and both the total load demand and fault current contributions are supplied solely by the integrated DG units. The network incorporates four inverter-based DG units, which represent the only sources feeding the system under islanded conditions. These DG units collectively supply the network and determine the available fault current levels, resulting in a highly constrained coordination environment due to their current-limiting behavior. The protection scheme consists of 29 DOCRs arranged to provide coordinated primary and backup protection throughout the meshed network. Relay settings, including CTR, pickup currents *I*p, are summarized in Table 2. Despite differences in CT ratios, a uniform pickup current of 300 A is adopted for all DOCRs, providing a consistent basis for the coordination process. Coordination performance is evaluated by applying three-phase faults at fifteen strategic locations (F1–F15) distributed across the network to assess selectivity under this severe operating mode.

**Table 2:** Pickup current and CT ratio data for DOCRs in the 30-bus system.

(DOCR)	CTR	IP
DOCR1	300/1	300
DOCR2	800/1	300
DOCR3	300/1	300
DOCR4	300/1	300
DOCR5	300/1	300
DOCR6-DOCR29	300/1	300

### Formulation of the relay coordination optimization problem

The relay coordination problem is formulated as a constrained nonlinear optimization problem^25,46^. The objective is to minimize the total operating time of primary relays under all considered fault scenarios while maintaining proper coordination between primary and backup relays.

#### Objective function

The objective function (OF) is defined as the minimization of the sum of primary relay operating times for all fault locations:

1 $$OF = min \Sigma T\_op,i,j \left( {for all primary relays i and faults j} \right)$$

#### Relay operating time

The operating time of each relay is calculated using the IEC standard inverse time–current characteristic, expressed as:

2 $$T_{op} = \frac{TMSi*\beta }{{\left( {\frac{{I_{fij} }}{{I_{pi} }}} \right)^{\alpha } - 1}}$$

where TMSᵢ = Time Multiplier Setting for relay I; Iᶠᵢⱼ = Fault current seen by relay i for fault j; Ipᵢ = Pickup current setting for relay I; α, β = IEC curve characteristic constants.

#### Coordination constraints

To ensure selective operation between primary and backup relays, a minimum CTI is imposed such that:

3 $$T_{op} ,backup - T_{op} ,primary \ge CTI = 0.3 \;seconds$$

where the CTI is set to 0.3 s^44^. In addition, practical limits are applied to the relay settings:

4 $$0.05 \le TMS\_i \le 1.1$$

5 $$Top, I \ge T min=0.2 seconds$$

#### Dual-setting relay formulation

For systems employing directional and DOCRs, separate settings are defined for forward and reverse fault directions^47^. The corresponding operating times are given by:

6 $${T}_{fwij}=\frac{TMSfwi*\beta }{{\left(\frac{{I}_{fwij}}{{I}_{pfwi}}\right)}^{\alpha }-1}$$

7 $${T}_{rvkj}=\frac{TMSrvk*\beta }{{\left(\frac{{I}_{frvkj}}{{I}_{prvk}}\right)}^{\alpha }-1}$$

The objective function is accordingly extended to include both forward and reverse relay operating times:

8 $$T={\sum }_{j=1}^{M}\left(\sum_{i=1}^{N}Tfwij+ \sum_{k=1}^{N}Trvkj\right)$$

The coordination constraint for dual-setting relays is expressed as:

9 $${T}_{rvkj} - {T}_{fwij}\ge CTI = 0.3 seconds$$

This formulation increases the dimensionality of the optimization problem, particularly in meshed networks, and highlights the need for robust optimization techniques capable of handling large constrained search spaces.

### Applied optimization algorithms

Three metaheuristic optimization algorithms are employed to solve the formulated coordination problem. The GA is adopted as a reference method due to its widespread application in relay coordination studies, while the WCA and the BB–BC algorithm are investigated as alternative approaches. All algorithms are implemented using identical objective functions, constraints, population sizes, and termination criteria to ensure a fair comparison. Short-circuit currents required for the optimisation process are obtained from ETAP simulations and imported into MATLAB, ensuring consistency between network modelling and optimization stages.

**Fig. 3:**
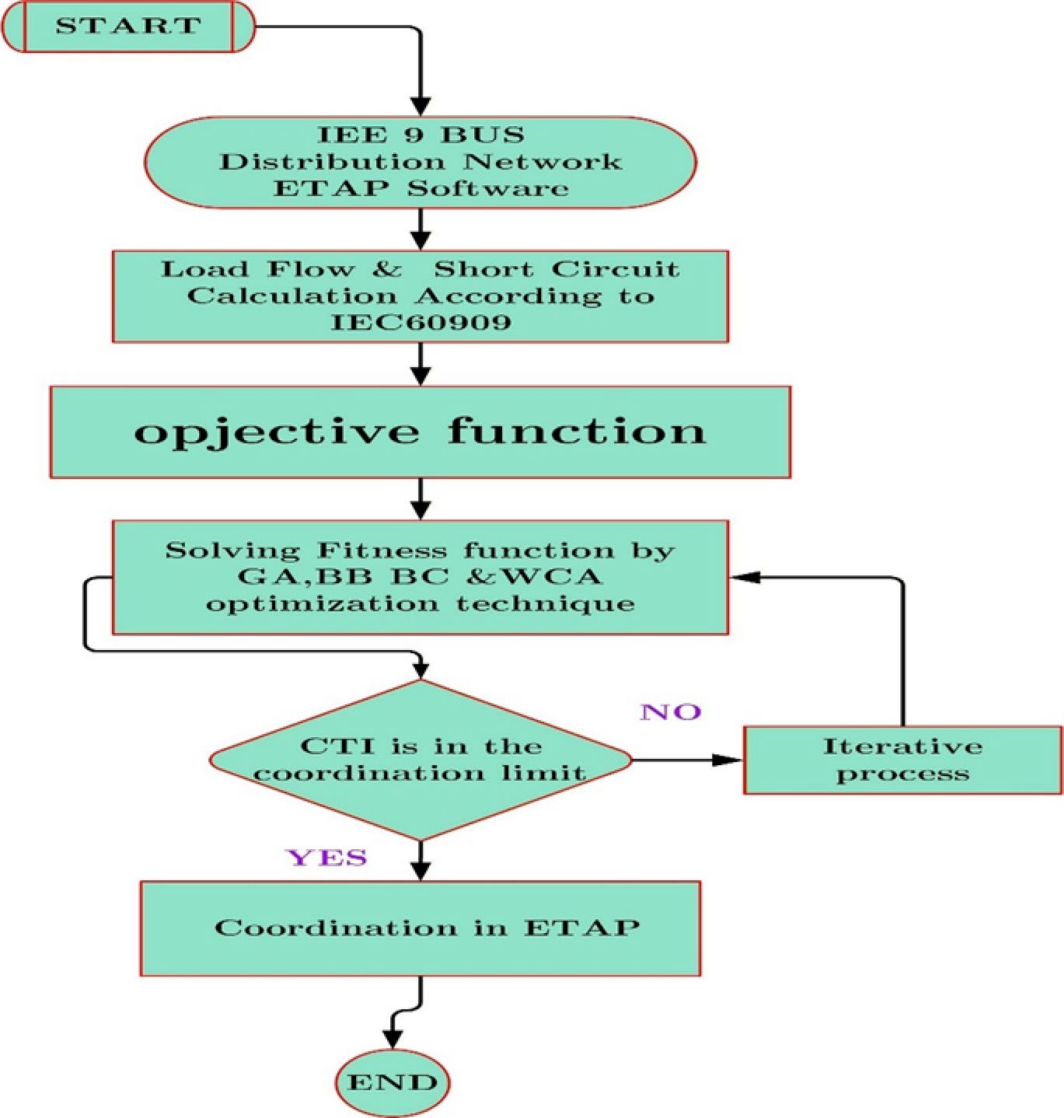
Flowchart of the relay coordination procedure for the 9-bus radial system.

The overall optimization procedure adopted in this study is illustrated in the flowchart shown in Fig. 3. Although the flowchart is presented in the context of the 9-bus system for clarity, the same optimization framework is identically applied to the 30-bus meshed system, with differences only in network data, number of relays, and coordination constraints.

## Results and discussion

### 9-Bus radial system results

The coordination performance of the proposed optimization approaches is first evaluated on the radial 9-bus distribution system under three operating modes.

#### Mode 1: Conventional grid-connected operation

Table 3 presents the relay coordination results obtained for the 9-bus system under conventional operation without DG. The primary and backup relay operating times together with the corresponding CTIs confirm that proper discrimination is maintained across all considered fault locations.

**Table 3:** GA-based relay coordination results for the radial 9-bus system under (Conventional—without DG).

Fault	Primary	Backup	T_primary (s)	T_backup (s)	CTI (s)
F1	OCR1	OCR2	0.0991	0.3981	0.2991
F2	OCR2	OCR3	0.3895	0.6887	0.2992
F3	OCR3	OCR4	0.6707	0.9698	0.2991
F4	OCR4	OCR17	0.9422	1.3837	0.4414
F5	OCR5	OCR6	0.0990	0.3981	0.2991
F6	OCR6	OCR7	0.3895	0.6707	0.2812
F7	OCR7	OCR8	0.7285	1.0275	0.2990
F8	OCR8	OCR17	1.0843	1.3837	0.2994
Total operating time	**11.3465**				

The CTI values are generally clustered around the required threshold of 0.3 s, with minor deviations observed in isolated cases. Despite these variations, overall selectivity is preserved and no miscoordination occurs. The total primary operating time obtained using GA is 11.35 s, reflecting fast and effective protection performance under radial modes. In addition to GA, both WCA and BB–BC achieved feasible relay coordination under this operating mode. The total operating times obtained by WCA (11.39 s) and BB–BC (12.09 s) are close to that of GA, confirming consistent performance under conventional radial network modes.

#### Mode 2: Grid-connected operation with full DG integration

**Fig. 4:**
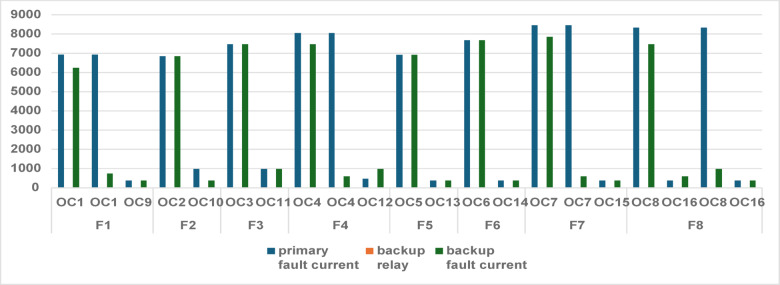
Fault current magnitudes observed by primary and backup relays in Mode 2.

The integration of all DG units significantly alters the short-circuit current distribution within the 9-bus radial system. Unlike the conventional operating mode, fault currents in Mode 2 are influenced by multiple injection points, resulting in bidirectional contributions and expanded protection participation. Consequently, additional relay pairs become involved in fault detection and coordination compared with Mode 1. Table 4 presents the primary and backup relay assignments together with the corresponding fault current magnitudes for all fault locations (F1–F8). The results confirm that DG integration changes the fault current distribution and increases the number of relays involved in fault detection, which directly increases the coordination burden. The wide spread between primary and backup fault current levels is further illustrated in Fig. 4.

**Table 4:** Primary and backup relay assignments with corresponding fault current magnitudes for each fault location in Mode 2 (Full DG integration).

Fault	Primary relay	I_primary (A)	Backup relay	I_backup (A)
F1	OCR1	6930	OCR2	6251
	OCR1	6930	OCR19	728
	OCR9	375	OCR18	375
F2	OCR2	6844	OCR3	6844
	OCR10	981	OCR9	375
F3	OCR3	7463	OCR4	7463
	OCR11	975	OCR10	975
F4	OCR4	8056	OCR17	7466
	OCR4	8056	OCR16	589
	OCR12	975	OCR11	975
F5	OCR5	6918	OCR6	6918
	OCR13	375	OCR20	375
F6	OCR6	7681	OCR7	7681
	OCR14	375	OCR13	375
F7	OCR7	8448	OCR8	7862
	OCR7	8448	OCR21	593
	OCR15	375	OCR14	375
F8	OCR8	8338	OCR17	7466
	OCR16	375	OCR21	589
	OCR8	8338	OCR12	969

The relay operating times and CTIs obtained using GA are summarized in Table 5. All relay pairs satisfy the required coordination constraint in Mode 2. For most fault scenarios, CTI values remain close to the 0.3 s threshold, indicating effective primary–backup discrimination under full DG integration. For example, the OCR8–OCR17 pair in Fault F8 exhibits a CTI of 0.895 s, which is significantly higher than the required 0.3 s threshold, whereas most other relay pairs remain closely clustered around the coordination boundary. Similarly, the OCR16–OCR15 pair records a CTI of 0.324 s, reflecting a slightly larger margin compared with the nominal coordination interval.

**Table 5:** Relay operating times and CTIs for the 9-bus system in Mode 2.

Fault	Primary	Backup	$T_{{{\mathrm{primary}}}}$ (s)	$T_{{{\mathrm{backup}}}}$ (s)	CTI (s)
F1	OCR1	OCR2	0.0953	0.3947	0.2995
	OCR1	OCR19	0.0953	0.3947	0.2994
	OCR9	OCR18	1.0181	1.3190	0.3010
F2	OCR2	OCR3	0.4886	0.7878	0.2992
	OCR10	OCR9	1.2654	1.5563	0.2999
F3	OCR3	OCR4	0.9570	1.2610	0.3040
	OCR11	OCR10	0.5213	0.8271	0.3058
F4	OCR4	OCR17	1.6560	1.9575	0.3014
	OCR4	OCR16	1.6560	1.9580	0.3019
	OCR12	OCR11	0.2234	0.5227	0.2993
F5	OCR5	OCR6	0.0953	0.3947	0.2994
	OCR13	OCR20	0.8676	1.1670	0.2995
F6	OCR6	OCR7	0.4923	0.7916	0.2994
	OCR14	OCR13	0.5623	0.8676	0.3052
F7	OCR7	OCR8	0.7708	1.0718	0.3010
	OCR7	OCR21	0.7708	1.0921	0.3213
	OCR15	OCR14	0.2624	0.5623	0.3000
F8	OCR8	OCR17	1.0621	1.9575	0.8953
	OCR16	OCR21	2.6388	2.9395	0.3007
	OCR16	OCR15	2.6388	2.9624	0.3245

**Fig. 5:**
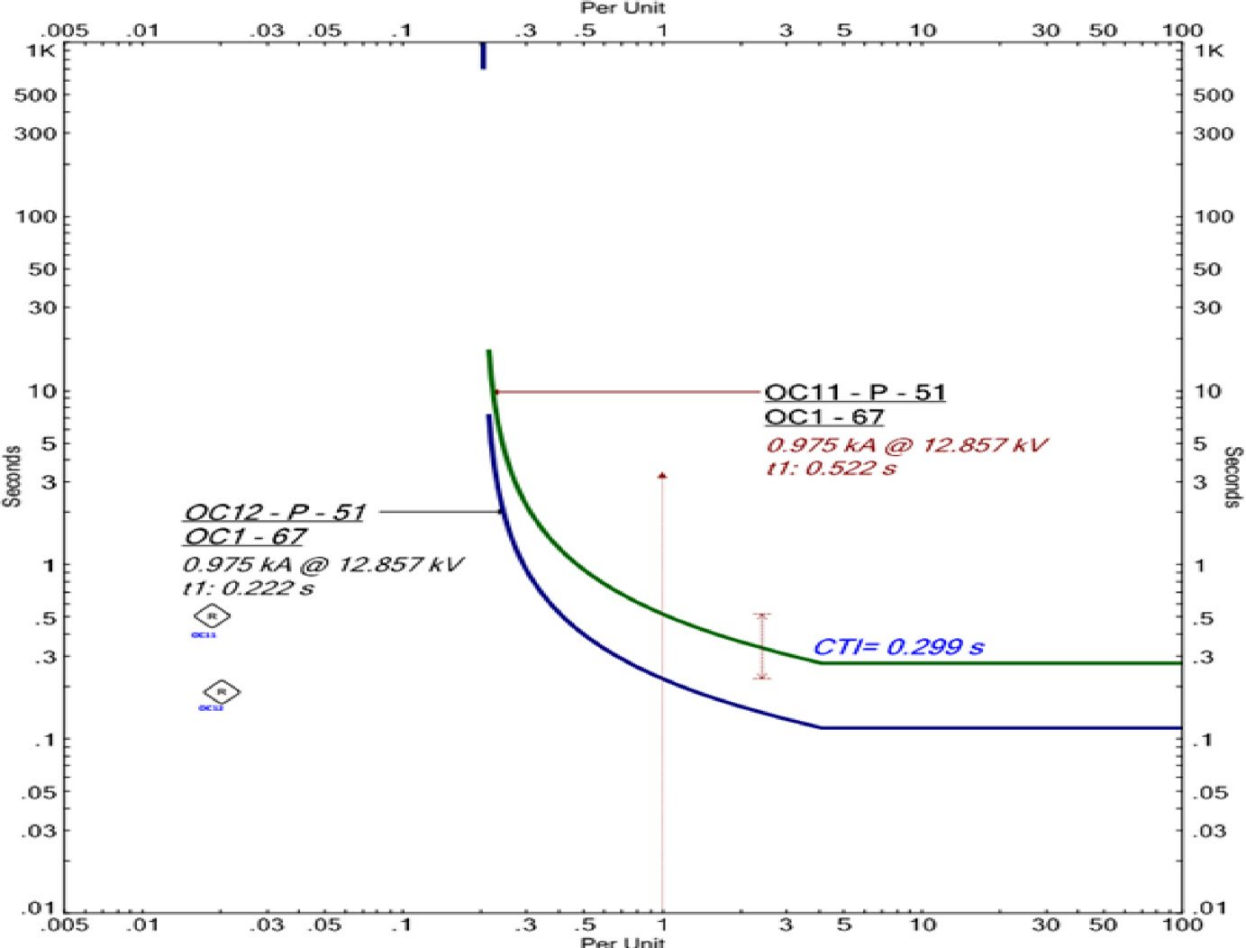
ETAP TCC curves illustrating relay coordination for fault scenario F4 in Mode 2.

A representative ETAP time–current characteristic (TCC) curve for fault scenario F4 is shown in Fig. 5, confirming correct coordination between the primary and backup relays in Mode 2.

The total operating time obtained in Mode 2 is 38.06 s for GA, compared with 70.22 s and 73.20 s for WCA and BB–BC, respectively. These results indicate that GA provides the fastest overall relay response under full DG integration, while the distribution of CTI values varies among the investigated optimization techniques.

#### Mode 3: Islanded operation

Islanded operation represents the most severe coordination condition for the 9-bus system. In this mode, the upstream grid is disconnected and fault currents are supplied solely by DG units. Consequently, fault levels are significantly reduced compared to grid-connected operation, which directly narrows the feasible coordination region and increases the optimization difficulty. Table 6 presents the operating times and CTIs obtained using GA for the islanded mode. for all fault locations (F1–F8). CTI values remain close to the required 0.3 s threshold, confirming that coordination is generally maintained despite the reduced fault currents. However, limited deviations appear under islanded modes. At F8, the OCR16–OCR15 pair records a CTI of 0.1093 s, which is below the coordination requirement and therefore represents a CTI violation. In addition, certain relay pairs exhibit excessively large CTI values (e.g., OCR10–OCR9 at F2 and OC16–OC21 at F8), reflecting delayed backup operation caused by reduced or uneven fault current distribution in the islanded network. Figure 8, 9, 10, 11 and 12 clear Statistical analysis of GA Mode 1,2, and 3 over 30 runs (a) boxplot with outliers, (b) histogram showing skewness, (c) convergence profile of best and mean trends.

**Table 6:** Presents the operating times and CTI values for all relay pairs in mode3.

Fault	Primary	Backup	${T}_{\mathrm{primary}}$ (s)	${T}_{backup}$ (s)	CTI (s)
F1	OCR1	OCR2	0.1830	0.4825	0.2995
	OCR1	OCR19	0.1830	0.4824	0.2994
F2	OCR9	OCR18	6.3405	6.6395	0.2990
	OCR2	OCR3	0.4795	0.7787	0.2992
F3	OCR10	OCR9	3.1928	6.3405	3.1477
	OCR3	OCR4	2.1969	2.4962	0.2993
F4	OCR11	OCR10	1.9150	2.2142	0.2992
	OCR4	OCR16	3.0644	3.3025	0.3090
	OCR12	OCR11	1.6212	1.9203	0.2992
F5	OCR5	OCR6	0.1731	0.4722	0.2991
	OCR13	OCR20	0.8602	1.1597	0.2995
F6	OCR6	OCR7	0.7066	1.0058	0.2991
	OCR14	OCR13	0.5608	0.8602	0.2995
F7	OCR7	OCR8	0.9998	1.2997	0.2999
	OCR7	OCR21	0.9998	1.2996	0.2998
F8	OCR15	OCR14	0.2613	0.5608	0.2995
	OCR16	OCR21	0.1520	1.3079	1.1559
	OCR16	OCR15	0.1520	0.2613	0.1093

**Fig. 6:**
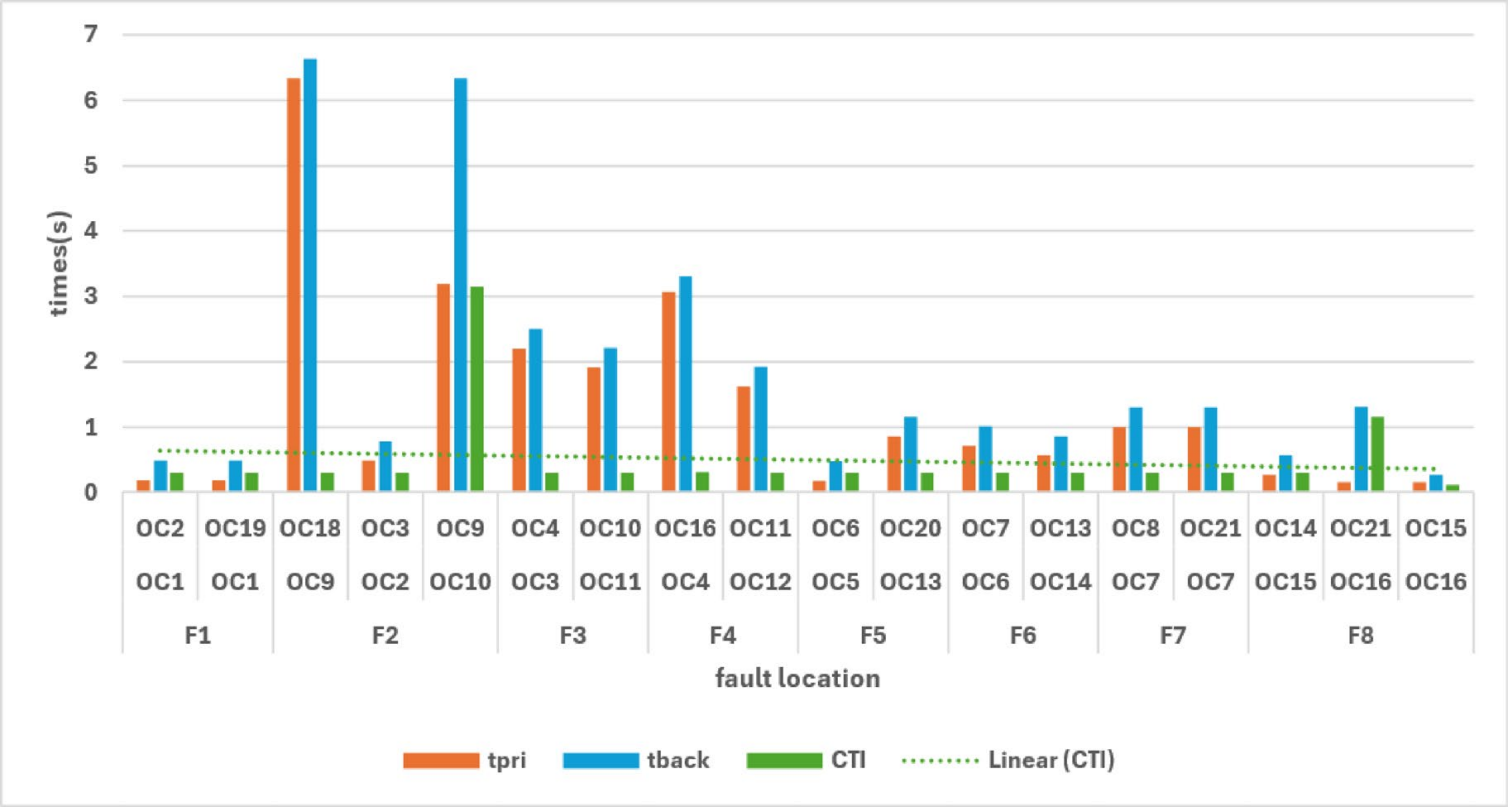
Fault current magnitudes observed by primary and backup relays in Mode 3.

Figure 6. illustrates the distribution of primary and backup operating times together with the CTI values. Most relay pairs cluster around the 0.3 s reference line, while only a few outliers are observed. These results highlight the increased coordination sensitivity in islanded operation due to the limited DG fault current contribution.

The total operating times in islanded mode are 74.79 s for GA, 89.35 s for WCA, and 144.00 s for BB–BC. GA achieves the lowest operating time; however, a CTI violation is observed under the reduced fault current conditions. WCA maintains coordination without violations, while BB–BC results in significantly higher operating time. A comprehensive comparison across all operating modes is provided in the next section.

### Comparative analysis across all operating modes

**Fig. 7:**
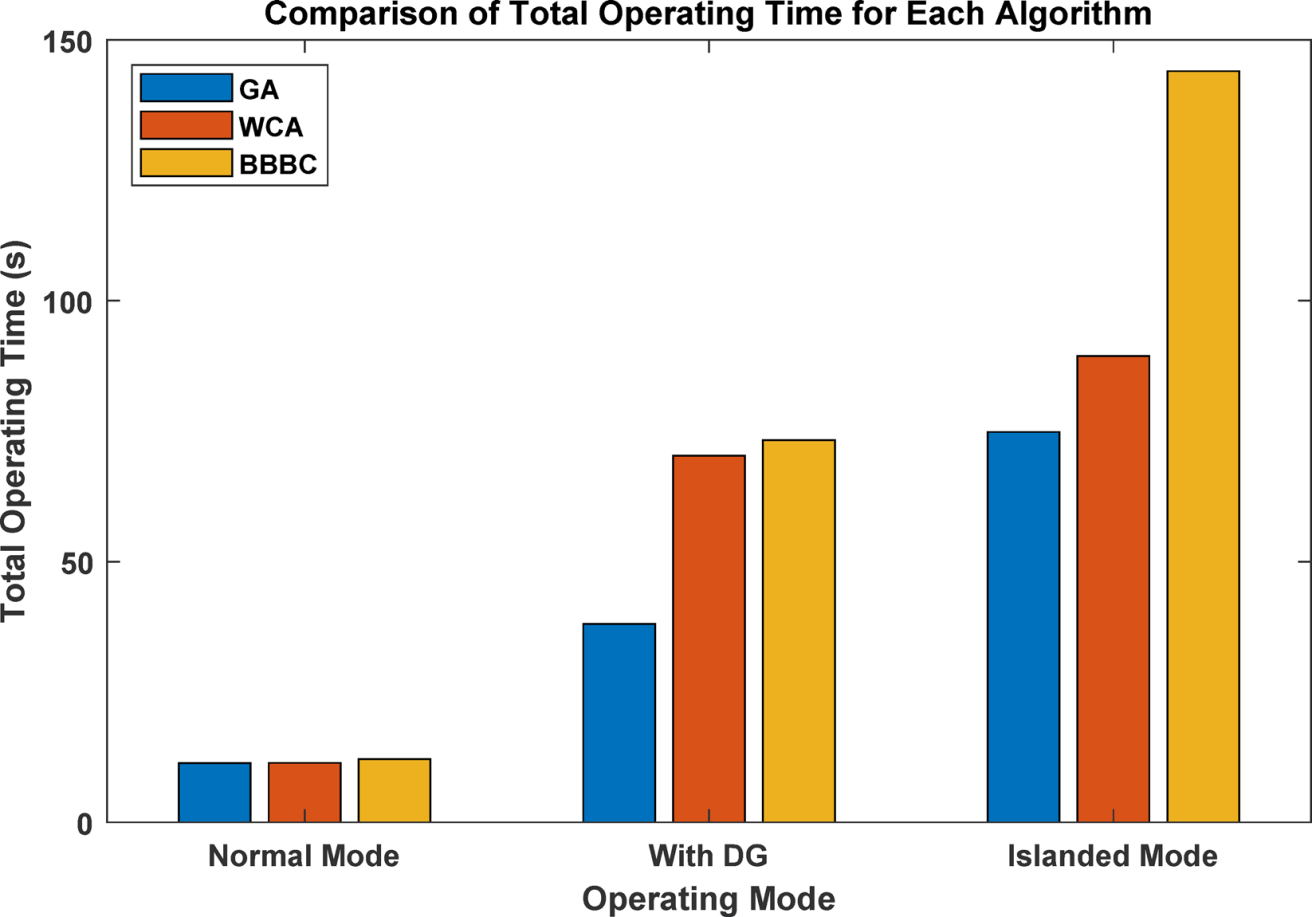
Comparison of total relay operating times obtained by GA, WCA, and BB–BC in conventional, full DG, and islanded operating modes.

**Fig. 8:**
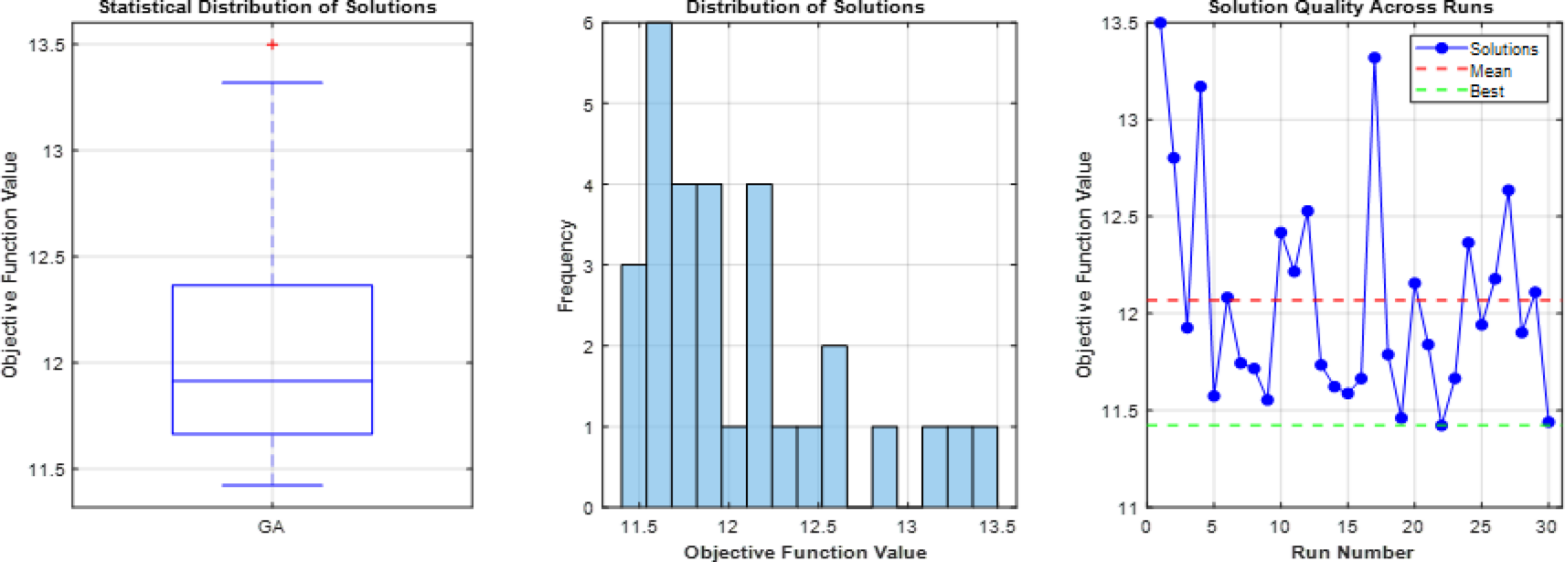
Statistical analysis of GA Mode 1 over 30 runs (**a**) boxplot with outliers, (**b**) histogram showing skewness, (**c**) convergence profile of best and mean trends.

**Fig. 9:**
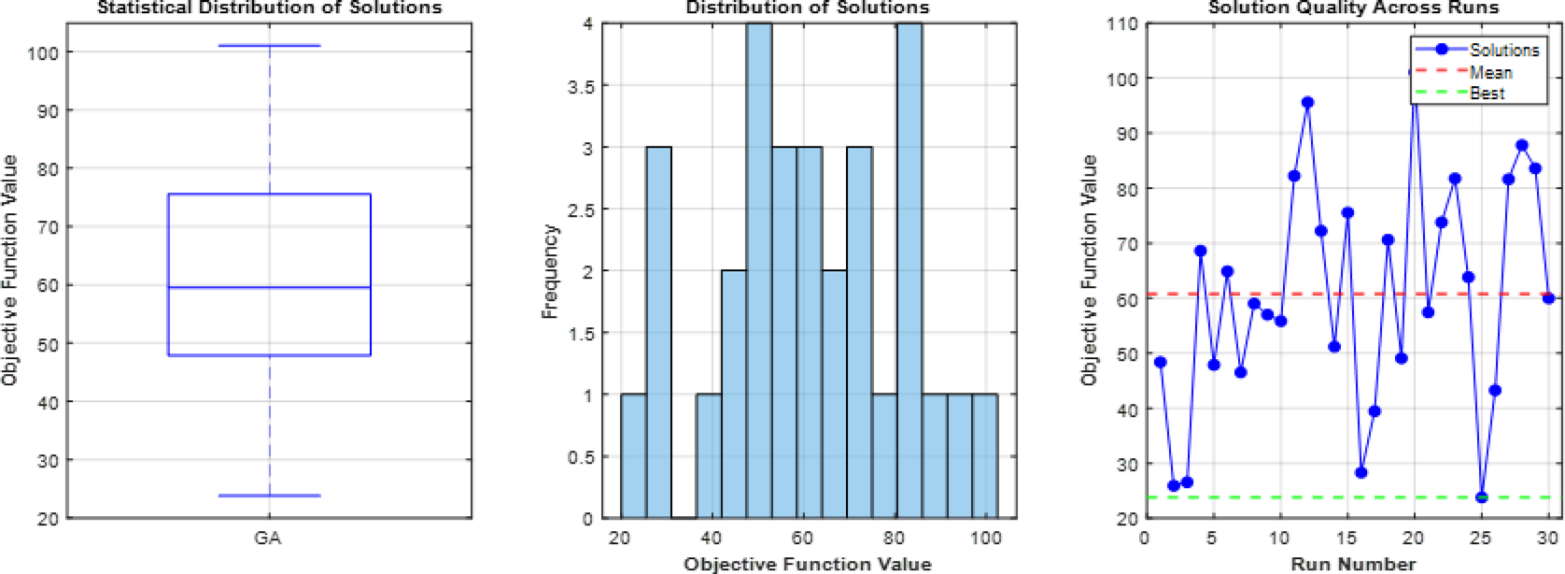
Statistical analysis of GA Mode 2 over 30 runs (**a**) boxplot with outliers, (**b**) histogram showing skewness, (**c**) convergence profile of best and mean trends.

**Fig. 10:**
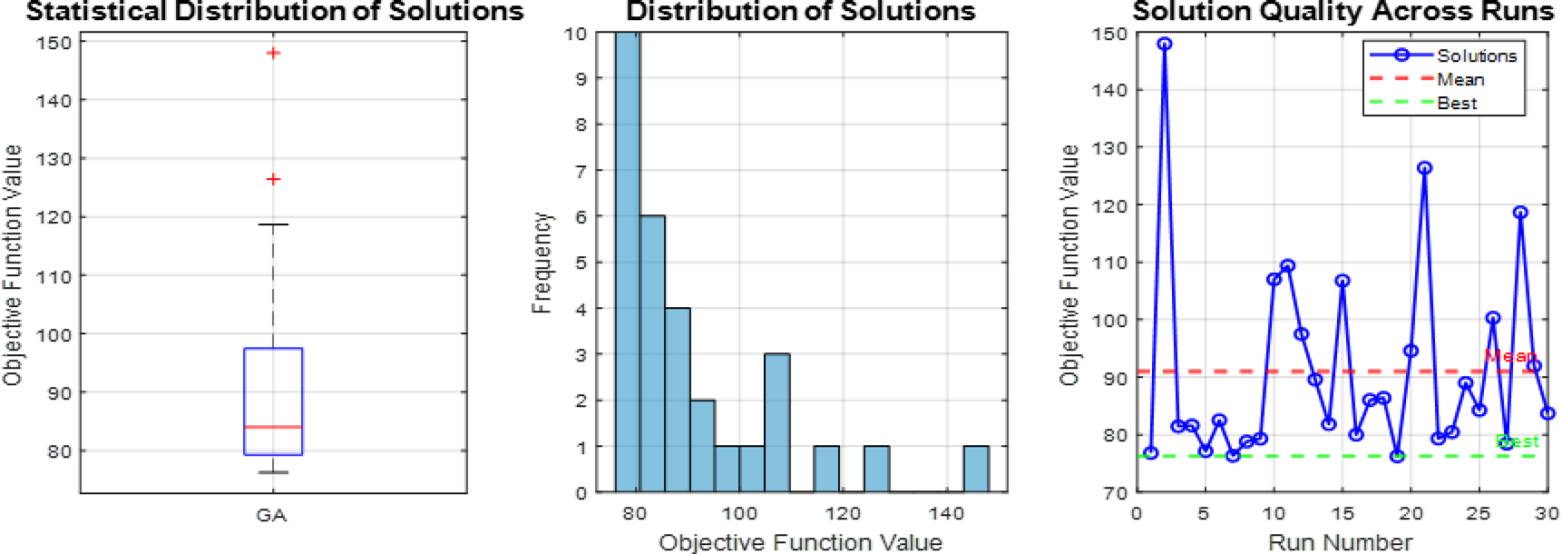
Statistical analysis of GA Mode 3 over 30 runs (**a**) boxplot with outliers, (**b**) histogram showing skewness, (**c**) convergence profile of best and mean trends.

**Fig. 11:**
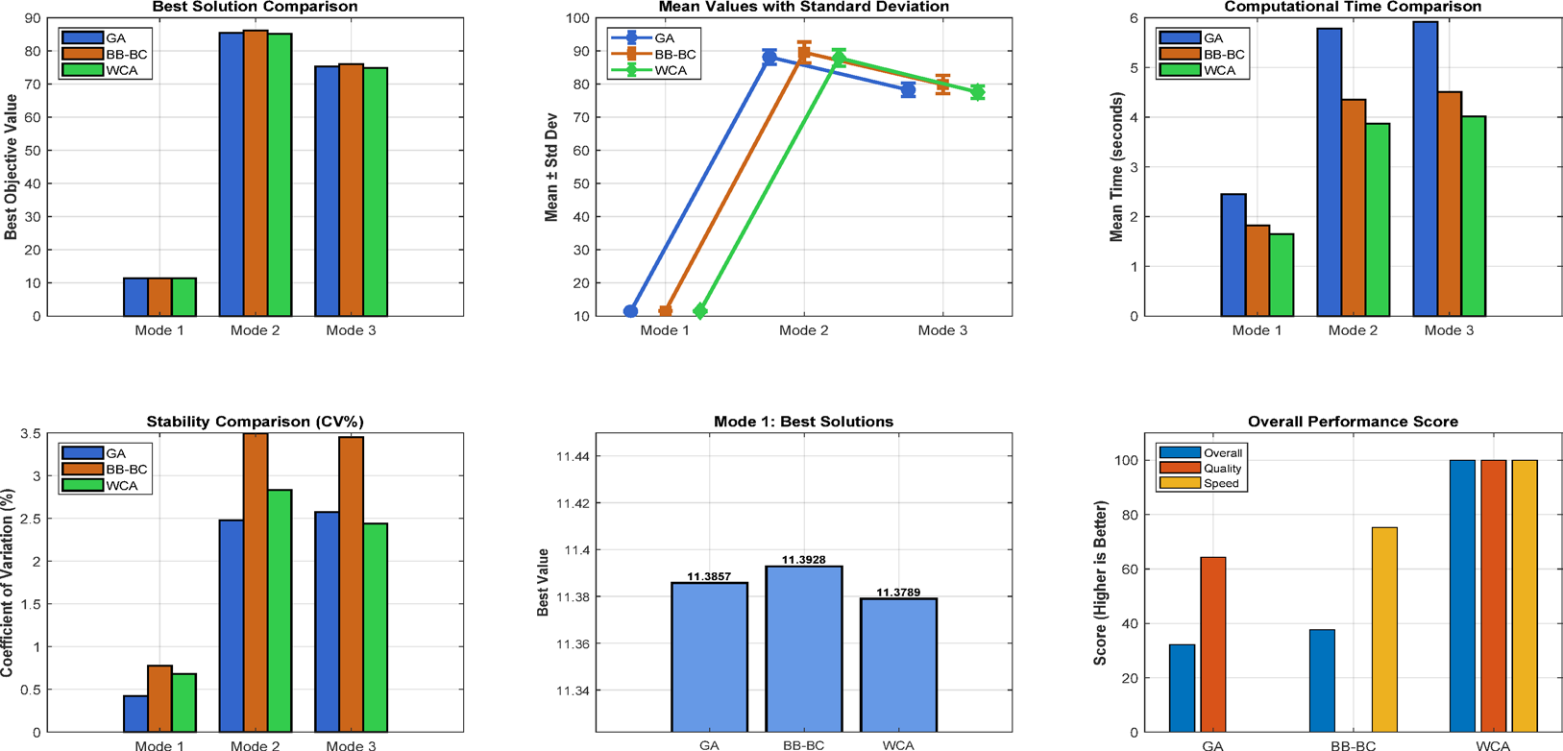
Statistical performance analysis.

The overall coordination performance of the 9-bus system was evaluated under three operating conditions: conventional operation without DG, full DG integration, and islanded mode. The optimized TMS values for all relays and the corresponding total operating times are summarized in Table 7, while the comparative total operating times are illustrated in Fig. 7. The statistical validation of the three optimization algorithms is presented through detailed analysis in Figs. 8, 9, 10, which show the performance of GA over 30 independent runs for Modes 1, 2, and 3, respectively. Each figure includes boxplots illustrating dispersion and outliers, histograms showi For Mode 1 (Fig. 8), WCA achieves the best objective function value (11.3789) with a coefficient of variation (CV%) of 0.681%, while GA demonstrates the highest stability (CV% = 0.424%). Similar trends are observed in Modes 2 and 3 (Figs. 9 and 10), where increased coordination complexity results in greater variability.ng distribution characteristics, and convergence profiles of best and mean trends. The overall statistical comparison among GA, BB–BC, and WCA is presented in Fig. 11. Figure 11a illustrates the objective function values across the three operating modes, Fig. 11b presents mean values with error bars (± 1 standard deviation) to quantify stochastic variability, and Fig. 11d shows the coefficient of variation for stability assessment. These results indicate that WCA provides a balanced performance in terms of solution quality, robustness, and computational efficiency across all investigated operating modes.

**Table 7:** Optimized TMS values and total operating times for GA, WCA, and BB–BC in the three operating modes of the 9-bus system.

Relay	Mode 1			Mode 2			Mode 3		
	GA	WCA	BB-BC	GA	WCA	BB-BC	GA	WCA	BB-BC
OCR1	0.050	0.051	0.058	0.050	0.050	0.057	0.050	0.050	0.050
OCR2	0.189	0.190	0.208	0.219	0.188	0.195	0.059	0.059	0.063
OCR3	0.286	0.286	0.299	0.456	0.413	0.436	0.120	0.120	0.115
OCR4	0.380	0.381	0.411	0.729	0.677	0.735	0.388	0.388	0.401
OCR5	0.054	0.054	0.080	0.050	0.050	0.052	0.050	0.050	0.050
OCR6	0.189	0.190	0.203	0.250	0.250	0.455	0.185	0.186	0.186
OCR7	0.285	0.286	0.303	0.430	0.429	2.010	0.297	0.460	0.341
OCR8	0.437	0.438	0.454	0.435	0.435	1.705	0.123	0.174	0.130
OCR9	–	–	–	0.210	0.194	0.282	1.218	1.616	1.560
OCR10	–	–	–	0.292	0.273	0.324	0.739	0.993	0.966
OCR11	–	–	–	0.192	0.174	0.189	0.639	0.830	0.772
OCR12	–	–	–	0.050	0.050	0.059	0.364	0.492	0.396
OCR13	–	–	–	0.167	0.165	0.229	0.167	0.165	1.265
OCR14	–	–	–	0.109	0.107	0.104	0.107	0.107	1.175
OCR15	–	–	–	0.050	0.050	0.058	0.050	0.050	0.679
OCR16	–	–	–	0.538	0.505	0.542	0.512	0.600	2.649
OCR17	0.414	0.415	–	1.342	1.260	1.963	0.000	0.000	0.000
OCR18	–	–	–	0.127	0.119	0.526	0.603	0.795	1.209
OCR19	–	–	–	0.074	0.074	1.093	0.091	0.092	0.821
OCR20	–	–	–	0.226	0.226	1.461	0.230	0.263	1.701
OCR21	–	–	–	0.485	0.458	0.501	0.204	0.290	0.462
Total Time	11.36	11.39	12.09	38.06	70.22	73.20	74.79	89.35	144.00

In conventional operation all algorithms achieved nearly identical and low operating times, confirming that relay coordination in a passive radial network can be efficiently handled by different optimization techniques. GA produced the lowest total operating time (11.36 s), with only minor differences compared to WCA and BB–BC. With full DG integration, coordination complexity increased due to bidirectional fault currents and multiple fault current contributions. Although GA produced the lowest total operating time (38.06 s), the solution did not fully satisfy coordination requirements for all relay pairs. In contrast, WCA and BB–BC maintained full coordination, with total operating times of 70.22 s and 73.20 s, respectively. Under this condition, WCA provided the most consistent coordination performance.

Islanded operation represents the most restrictive coordination scenario due to reduced short-circuit levels and limited fault current contribution from DG units. Under these conditions, all algorithms exhibited increased operating times. GA achieved a total operating time of 74.79 s but showed coordination sensitivity under strict constraints. WCA preserved coordination feasibility with a total operating time of 89.35 s, whereas BB–BC resulted in 144.00 s, reflecting difficulty in navigating the severely constrained solution space. Table 8 presents the comprehensive statistical comparison of GA, BB–BC, and WCA across the three operating modes based on 30 independent runs with 200 iterations each. Mode 1 involves 9 decision variables (basic configuration), while Modes 2 and 3 involve 21 decision variables due to DG integration. The reported metrics include best and worst objective values, mean performance, standard deviation (Std. Dev.), coefficient of variation (CV%), and mean computational time. Bold values indicate superior performance in each category.The overall statistical analysis shown in Fig. 11 further highlights the comparative behavior of the algorithms. As illustrated in Fig. 11a, WCA consistently achieves competitive objective function values across all operating modes (11.3789, 85.1263, and 74.8924 for Modes 1, 2, and 3, respectively). Figure 11b presents the mean values with error bars (± 1 standard deviation), confirming the robustness of the algorithms. The coefficient of variation analysis in Fig. 11d indicates that GA exhibits the highest stability in Modes 1 and 2 (CV% = 0.424% and 2.478%, respectively), while WCA demonstrates strong stability in Mode 3 (CV% = 2.440%). Overall, GA demonstrates strong objective minimization capability under moderately constrained conditions, whereas WCA provides a balanced trade-off between solution quality, stability, and coordination feasibility across all investigated scenarios. BB–BC remains stable but becomes less efficient as coordination complexity increases.

**Table 8:** Comprehensive statistical analysis of optimization algorithms for 9-bus relay coordination problem.

Mode	Algorithm	Num Vars	Best	Worst	Mean Val	Std Dev	CV percent
Mode 1	GA	9	11.3857	11.5623	11.4422	0.0485	0.423869536
Mode 1	BB-BC	9	11.3928	11.7456	11.5187	0.0895	0.776997404
Mode 1	WCA	9	11.3789	11.6895	11.4966	0.0782	0.680201103
Mode 2	GA	21	85.4726	92.8406	88.1564	2.1846	2.478095748
Mode 2	BB-BC	21	86.1246	95.7823	89.5478	3.1285	3.493664836
Mode 2	WCA	21	85.1263	93.4587	87.8925	2.4879	2.830616947
Mode 3	GA	21	75.3125	82.5679	78.2454	2.0159	2.576381487
Mode 3	BB-BC	21	76.0489	84.9236	79.8322	2.7563	3.452616864
Mode 3	WCA	21	74.8924	81.3456	77.5648	1.8923	2.439637568

### Results for the 30-bus distribution system

The 30-bus mesh system implements DOCRs to handle bidirectional fault currents inherent in meshed topologies with distributed generation. This configuration represents modern active distribution networks with multiple power flow paths and complex protection requirements. Fault scenarios F1-F15 are strategically distributed throughout the network to evaluate comprehensive coordination performance under diverse conditions. Each fault location tests different relay pairs in both forward and reverse directions. Table 9. summarizes the optimized relay operating times obtained using GA, BB-BC, and WCA for all fault locations. The total operating times are 40.63 s for GA, 39.10 s for BB-BC, and 38.13 s for WCA. The results indicate that all three algorithms succeed in achieving feasible coordination across the investigated scenarios. However, WCA provides the lowest cumulative operating time, followed closely by BB-BC, while GA yields a slightly higher total delay.

**Table 9:** Optimization results for 30-bus mesh system.

Fault	Fault current (A)	CMS	Relay	TMS (GA)	TMS (WCA)	TMS (BB-BC)
F1	12,954	25.91	DOCR1-F	0.05	0.04	0.04
	2527	3.16	DOCR2-R	1.52	0.25	0.25
	2527	8.42	DOCR3-F	0.46	0.45	0.45
F2	10,428	20.86	DOCR2-F	0.57	0.48	0.47
	3655	4.57	DOCR9-R	0.70	0.53	0.53
	2088	6.96	DOCR5-F	0.69	0.66	0.66
F3	2925	9.75	DOCR4-F	0.42	0.41	0.41
	7375	24.58	DOCR5-F	0.41	0.30	0.30
	6177	7.72	DOCR6-R	0.62	0.41	0.51
F4	3202	10.67	DOCR6-F	0.07	0.07	0.07
	9416	31.39	DOCR7-F	0.71	0.48	0.48
	2085	6.95	DOCR8-R	1.12	0.68	0.68
F5	3645	12.15	DOCR10-F	0.07	0.06	0.06
	1226	4.09	DOCR11-R	0.27	0.26	0.26
F9	4360	14.53	DOCR16-F	0.24	0.38	0.38
	2980	9.93	DOCR17-F	0.07	0.11	0.11
	850	2.83	DOCR23-R	0.61	0.31	0.31
F11	6661	22.20	DOCR20-F	0.05	0.04	0.04
	4700	15.67	DOCR21-F	0.06	0.06	0.06
	1645	5.48	DOCR22-R	0.27	0.27	0.27
F15	5314	17.71	DOCR27-F	0.06	0.05	0.05
	2270	7.57	DOCR24-R	0.26	0.25	0.25
Total operating time				**40.63 s**	**38.13 s**	**39.10 s**

For each fault scenario, the relay operating in the forward direction (F) acts as the main clearing device for that fault path, while the corresponding reverse (R) setting of adjacent relays provides the required time-graded backup in the opposite direction. The optimized results confirm proper separation between forward and reverse operating times, ensuring secure discrimination without directional mis operation. Although the performance differences among the algorithms are moderate, WCA achieves the lowest cumulative operating time while maintaining stable coordination across all directional relay pairs. The close proximity of the obtained totals indicates that the dual-setting formulation increases the dimensionality of the search space but remains tractable for the investigated metaheuristic techniques.

## Practical deployment considerations and limitations

Although the proposed optimization framework demonstrates effective coordination performance under the investigated operating modes, several practical considerations must be acknowledged before real-world deployment. First, the relay TMS are treated in this study as continuous decision variables, whereas practical digital relays implement discrete setting steps with limited resolution. Consequently, the optimal values obtained from the metaheuristic algorithms may require rounding to the nearest available setting, which can slightly alter CTIs. In practical applications, a post-optimization verification stage is therefore necessary to ensure that discretized settings preserve coordination feasibility. Second, the short-circuit currents used in this study are calculated according to IEC 60909 under defined operating modes. In actual distribution networks, fault current levels vary with system topology, DG penetration level, and generation dispatch. Inverter-based distributed generators, in particular, typically provide limited and controlled fault current contributions, especially during islanded operation. These variations may narrow coordination margins and increased sensitivity to parameter uncertainty. For this reason, practical implementation may require conservative CTI margins greater than the nominal 0.3 s adopted in this work. Furthermore, non-ideal hardware characteristics are not explicitly modeled in the optimization framework. Current transformer saturation, measurement errors, breaker mechanical operating time variability, and relay processing delays can influence effective clearing times in practice. These factors may reduce the effective discrimination margin between primary and backup relays, particularly under low fault current conditions. Finally, while the proposed methodology is validated on 9-bus and 30-bus benchmark systems, large-scale practical distribution networks may involve significantly higher numbers of relays and coordination constraints. In such cases, computational burden and convergence reliability become important considerations, and parallel or hybrid optimization strategies may be required. Despite these limitations, the presented framework provides a structured and extensible foundation for relay coordination in DG-integrated distribution systems and can be further enhanced to incorporate discrete settings, uncertainty modeling, and large-scale implementation requirements.

## Conclusion

This study investigated overcurrent relay coordination in DG-integrated distribution networks under different operating modes. The results show that coordination performance becomes increasingly sensitive to fault current distribution as DG penetration increases, particularly under islanded operation where fault levels are significantly reduced. The analysis confirms that achieving reliable coordination in DG-dominated networks requires optimization techniques with strong robustness and effective constraint-handling capability. As coordination margins become narrower, maintaining feasibility across different operating modes becomes more critical than aggressive minimization of relay operating times. Overall, the findings emphasize the need for reliable and robust coordination strategies for modern active distribution networks and provide useful insight into the suitability of metaheuristic optimization approaches for practical protection applications.

In the radial 9-bus system, the GA achieves the lowest total operating time under conventional grid-connected operation (11.36 s), followed closely by WCA (11.39 s) and BB–BC (12.09 s). However, under DG-integrated conditions (Mode 2), GA records 38.06 s, while WCA and BB–BC result in 70.22 s and 73.20 s, respectively. Under islanded operation (Mode 3), GA achieves 74.79 s, WCA results in 89.35 s, and BB–BC reaches 144.00 s, reflecting the increased coordination difficulty under reduced fault current levels.

In the islanded 30-bus system, WCA attains the minimum total operating time, followed by BB–BC, while GA yields slightly higher operating times but maintains stable coordination behavior. Statistical analysis over 30 independent runs confirms consistent performance, with GA showing the lowest variability in Mode 1 (CV% = 0.424%) and WCA demonstrating strong robustness in Mode 3 (CV% = 2.440%).

## Data Availability

The datasets analyzed during the current study are available from the corresponding author on reasonable request.
